# Can Digital Finance Promote Comprehensive Carbon Emission Performance? Evidence from Chinese Cities

**DOI:** 10.3390/ijerph191610255

**Published:** 2022-08-18

**Authors:** Hanhua Shao, Jixin Cheng, Yuansheng Wang, Xiaoming Li

**Affiliations:** 1 Research Center of the Central China for Economic and Social Development, Nanchang University, Nanchang 330031, China; 2 School of Business, Central South University, Changsha 410083, China; 3 Antai College of Economics and Management, Shanghai Jiao Tong University, Shanghai 200030, China; 4 Center for Economic Research, Shandong University, Jinan 250100, China

**Keywords:** digital finance, carbon emission performance, low-carbon development, Chinese cities

## Abstract

Improving urban comprehensive carbon emission performance (CCEP) is the inevitable choice for China’s low-carbon development. With the continuous integration of digital technology and financial elements, the development of urban digital finance has also been significantly improved. To further explore the impact of urban digital finance on urban low-carbon development, using the data of 281 cities in China from 2011 to 2019, this paper firstly evaluates the urban CCEP, and further empirically investigates how digital finance influences CCEP. The empirical results show that: (1) Digital finance significantly improves the urban CCEP, and after conducting robustness tests and addressing the endogeneity issue, the above conclusion is robust. (2) For the sub-indicators, there is a U-shaped relationship between the coverage breadth of digital finance and CCEP. Moreover, the improvement of usage depth and digital support services could promote CCEP. (3) The channel tests indicate that digital finance improves the CCEP mainly by promoting green technology innovation and the development of urban tertiary industry. Meantime, digital finance has a stronger impact on improving CCEP in cities with more developed traditional finance, and the positive effect is significant in non-old industrial base cities and a two-control zone. Finally, this paper puts forward relevant policy suggestions.

## 1. Introduction

Dealing with climate change has become an urgent task for all countries around the world. According to the IPCC report, realizing substantial cuts in carbon emissions by 2030 and net-zero carbon emissions by 2050 could limit global warming to 1.5 °C, hence avoiding the irreversible negative impacts on ecosystems and human society. Given the importance of reducing carbon emissions in dealing with climate change, China, as a major carbon emitter, has committed to a carbon peak by 2030 and carbon neutrality by 2060. Hence, it is of great significance to investigate how to promote the carbon peak and carbon neutrality in China.

Considering the strong relationships between economic development, carbon emissions, and energy consumption [1,2,3], it is important to reasonably evaluate the regional comprehensive carbon emission performance (CCEP). CCEP represents the low-carbon development level, that is, fully considering the regional economic development and resource allocation capacity in the process of evaluating the regional CO_2_ emission level. Promoting comprehensive carbon emission performance (namely, increasing resources utilization rate alongside economic development to reduce CO_2_ emissions) could contribute to the carbon peak and carbon neutrality, and it is also the inevitable choice for China’s low-carbon development. Numerous studies have investigated how to promote carbon emission performance from the perspective of environmental policies [4,5,6], city characteristics [7], urbanization [8,9], FDI [10,11], and innovation [12,13]. The importance of finance in reducing carbon emissions and promoting efficiency has also been a widely discussed topic in recent years. Demertzis, et al. [14], Jalil and Feridun [15], and Acheampong [16] find that financial development contributes to reducing carbon emissions through technological development and corporate governance promotion. However, financial development may have a negative influence on carbon emission, since it enables households and enterprises to obtain cheaper credit, thus forcing them to purchase household equipment or machines that are energy-consuming [16,17,18]. Overall, among existing studies, the conclusions about the influence of financial development on carbon emission are conflicting.

The Chinese traditional financial system is insufficient as the resource allocation is influenced by the government, which excludes many enterprises and households from the financial system [19]. The shortcoming of China’s financial system fosters the birth and development of new financial models to some extent. In recent years, through online payment, big data, and cloud computing technology, digital finance improves people’s well-being by providing more inclusive financial support. Numerous studies have investigated the economic consequences of digital finance from the perspective of enterprise financial behavior [20], household consumption [21], financial stability [22,23], entrepreneurship [24], and innovation [25,26,27]. Their overall conclusion is that digital finance can supplement the traditional financial system to a large extent. Moreover, some studies also focus on exploring the impact of digital financial inclusion on the environment and regional sustainable development. Ullah, et al. [28] found that financial inclusion had a positive impact on the sustainable development of countries in the one belt and road initiative (OBRI) region; and based on data from 103 countries, Renzhi and Baek [29] found that digital financial inclusion can mitigate CO_2_ emissions. On the contrary, Ozturk and Ullah [30] found that digital financial inclusion can significantly boost the economic growth of countries in the OBRI region, but decrease environmental quality. Le, et al. [31] found that financial inclusion led to increased CO_2_ emissions based on 31 economies in Asia. Nevertheless, few studies have investigated how digital finance influences carbon emission performance.

Compared with the traditional finance, digital finance performs better in reducing information asymmetry and identifying borrowers’ default risks [22,32]. Therefore, under the model of digital finance, green innovative projects and small and micro-sized enterprises in the tertiary industry, which are generally excluded from the traditional financial system, could more easily obtain financial support. Extensive evidence shows that green innovation is positively correlated with carbon emissions [33,34], while the tertiary industry share is negatively correlated with carbon emissions [35]. Hence, we hypothesize that digital finance could promote comprehensive carbon emission performance.

Using city-level data from China’s mainland, we investigate how digital finance influences comprehensive carbon emission performance. We first introduce a super-SBM method based on a total factor analysis framework to assess the CCEP of China’s cities, which can effectively evaluate the urban green and low-carbon development level considering economic factors and avoid the influence of censored data on empirical analysis. In addition, our estimation results show that the digital finance index is positively correlated with carbon emission performance. After conducting a set of robustness tests and addressing the endogeneity issue, the above conclusion is robust. Our channel tests indicate that digital finance improves urban CCEP by promoting green innovation and the development of the tertiary industry. We finally investigate how the urban development characteristics and the degree of policy control influence the relationship between digital finance and CCEP. Our results show that digital finance has a stronger impact on CCEP in cities with more developed traditional finance systems, and the positive effect is significant in non-old industrial base cities and a two-control zone.

We contribute to the existing literature in the following aspects. First, we provide more reliable results on how digital finance influences carbon emission performance. Different from the previous studies, we focus on urban low-carbon development and empirically examine how digital finance influences carbon emission performance based on city-level data. Additionally, we conduct more comprehensive channel tests, endogeneity tests, and cross-sectional tests. Our above empirical framework enables us to precisely identify the causal relationship between digital finance and carbon emission performance. Second, we also present the dynamic evolution of comprehensive carbon emission performance of China’s 281 cities from 2011 to 2019, which could help the policymakers comprehend the characteristics of urban CCEP and know the gains and losses in China’s low carbon development. Thirdly, our results contribute to reconciling how financial development influences carbon emission performance. Existing studies show conflicting conclusions on how financial development influences carbon emissions. In this paper, we reveal that, when combined with digital technology, financial development could promote urban CCEP.

Furthermore, in Section 2, we further analyze the theoretical channels about how digital finance influences CCEP and propose several hypotheses. In Section 3, we introduce the methodology and present the descriptive statistics of our data. Then, in Section 4, we report and discuss our estimation results. We finally discuss our conclusions and implications in Section 5.

## 2. Theoretical Analysis and Theoretical Development

### 2.1. Digital Finance, Green Innovation and CCEP

Urban green innovation is crucial in promoting carbon emission performance. Extensive evidence shows that green innovation contributes to reducing carbon emissions and promoting output efficiency [33,34,36,37]. Stable and sufficient funding is one of the most important prerequisites in green innovation [38]. However, China’s financial system is bank-oriented [39] and the financial support from banks to green innovative activities is relatively un-efficient. First, innovative activities are accompanied by huge sunk costs, high output uncertainty, and positive externalities [40]. If an enterprise fails in its innovative activities, the bank may lose all its principles. If the enterprise succeeds, the bank will only obtain a fixed amount of interest, which is not commensurate with the risk it takes [41]. Consequently, banks are generally not willing to support green innovative activities. Second, banks often evaluate the profitability and debt-paying ability of enterprises by financial reports, and they generally do not have the skills to evaluate the value and risk of projects with few fixed assets and high uncertainty [40,42]. Hence, green innovative projects are often excluded from the financial service of banks.

Digital finance could reduce information asymmetry and trading costs through digital technology [32,39]. Compared with traditional risk management technology, big data risk management technology performs better in predicting borrowers’ default risks [22]. By using big data risk management technology, digital finance could provide financial support to green innovative projects according to their prediction of default risk, which could alleviate the financial constraints of green innovative projects in the traditional financial system. Additionally, by breaking the time–space boundaries and promoting the automation of the business process, digital finance promotes the convenience and flexibility of the financial service targeted at innovative projects [14]. Therefore, digital finance performs better in satisfying the heterogeneous financial demand of green innovative enterprises.

Based on the above analysis, we propose the following hypothesis:

**Hypothesis** **1** **(H1).** *Digital finance could promote CCEP by promoting green innovation*.

### 2.2. Digital Finance, Industrial Structure, and CCEP

The industrial structure also significantly influences carbon emission performance. Compared with other industries, the tertiary industry is less energy-intensive and could generate more added value [43]. Existing studies have revealed that the secondary industry share is positively correlated with carbon emissions [44,45], while the tertiary industry share is negatively correlated with carbon emission [35].

Digital finance could foster the development of the tertiary industry. The internet and information technology reduce the searching cost, evaluation cost, and trading cost of customers, and hence changes the traditional business [46,47]. The development of digital finance enables merchants and consumers to complete the trading process online using mobile payments, thus improving customers’ paying experience. Additionally, instead of managing risks by requiring a mortgage, digital finance controls and manages the risk according to big data consisting of customers’ trading behavior, which could foster their intertemporal consumption. Digital finance fosters the development of numerous new business models, such as E-commerce, online ride-sharing services, and bike-sharing by online payment technology. Therefore, digital finance contributes to generating more consumer demand for services.

Additionally, small and micro-sized enterprises, especially those that belong to the tertiary industry, benefit a lot from digital finance. In China’s traditional financial system, banks are not willing to provide small and micro-sized enterprises with loans due to the requirements of risk management, making these enterprises exposed to severe financial constraints [48]. By evaluating and managing risks through big data technology, digital finance could evaluate borrowers’ default risks more accurately than traditional finance [22]. Therefore, digital finance could help small and micro-sized enterprises belonging to the tertiary industry to obtain credit, thus fostering the development of the tertiary industry.

Therefore, we propose the hypothesis:

**Hypothesis** **2** **(H2).** *Digital finance could promote CCEP by fostering the development of the tertiary industry*.

## 3. Empirical Methods and Data Description

In this section, the global production possibilities set and super slacks-based measure (super-SBM) model have collaborated in Section 3.1, which is employed to evaluate the CCEP of China’s cities. Section 3.2 describes the empirical econometric model. The data descriptions are presented in Section 3.3.

### 3.1. Global Production Possibilities set and Super-SBM Model

To accurately evaluate the urban green and low-carbon development level considering various inputs and economic outputs factors, here we introduced a super-SBM DEA model, which can effectively assess the ratio of the ideal value to the actual value of input variables, economic output variables, and CO_2_ emissions [49,50]. Assume that the number of DMUs is *N* and there are three categories of variables; namely, inputs, desirable, and undesirable outputs, which are represented as three vectors, viz., $X=\left[x_{1},\ldots ,x_{N}\right]\in R^{M\times N}$, $Y=\left[y_{1},\ldots ,y_{N}\right]\in R^{K\times N}$, $C=\left[c_{1},\ldots ,c_{N}\right]\in R^{H\times N}$, respectively, and *M*, *K*, *H* denote the numbers of the variables. Because this paper focuses on urban comprehensive carbon performance, the undesirable outputs in this paper only include urban CO_2_ emissions, and *H* = 1. Meantime, following Pastor and Lovell [51] and Zhu, et al. [52], the global production possibilities sets are further introduced to measure the urban CCEP, which can be defined as follows:

(1) $$P^{G(t)}(x^{G(t)})=\left\{(y^{G(t)},c^{G(t)}):x^{G(t)}can\begin{array}{c} produce(y^{G(t)},c^{G(t)}) \end{array}\right\}$$

where *t* represents the year. *P^G^* presents the global production possibility set.

Following Huang, et al. [49] and Tone [53], the carbon emission performance of sample intervals could be obtained by solving the following super-SBM model:

(2) $$\begin{array}{l} P^{G}(\theta^{*})=\min\frac{1-\frac{1}{M}\int_{m=1}^{M}\frac{s_{m}^{-}}{x_{mo}}}{1+\frac{1}{K+H}\left(\int_{k=1}^{K}\frac{s_{k}^{y}}{y_{ko}}+\int_{h=1}^{H}\frac{s_{h}^{c}}{c_{ho}}\right)}\\ \left\{s.t.\begin{array}{c} x_{o}-\int_{j=1,\neq o}^{N}\lambda_{j}x_{j}+s^{-}\geq 0;-y_{o}+\int_{j=1,\neq o}^{N}\lambda_{j}y_{j}+s^{y}\geq 0;c_{o}-\int_{j=1,\neq o}^{N}\lambda_{j}c_{j}+s^{c}\geq 0\\ 1-\frac{1}{K+H}\left(\int_{k=1}^{K}\frac{s_{k}^{y}}{y_{ko}}+\int_{h=1}^{H}\frac{s_{h}^{c}}{c_{ho}}\right)\geq \varepsilon ;\lambda_{j},s^{-},s^{y},s^{c}\geq 0;\sum_{j=1,\neq o}^{N}\lambda_{j}=1 \end{array}\right\} \end{array}$$

where $\theta^{*}$ is the performance value of DMU*_o_*. $s^{-}$, $s^{y}$, $s^{c}$ are the slacks in inputs, desirable outputs, and CO_2_ emissions. $\lambda_{j}$ is the intensity variable. The term ε is non-Archimedean infinitely small. To consider the scale characteristics of urban carbon emission, the constraints of variable returns to scale were further added to the performance evaluation model. At the same time, it should be emphasized that to obtain the performance value, the fractional program must be transformed into a linear programming [49,54]. In addition, different from the standard SBM model, the carbon emission performance value evaluated based on the super model can be greater than 1. At the same time, a larger value reflects a higher comprehensive carbon performance.

### 3.2. Econometric Models

The basic econometric model is defined as follows:

(3) $$CCEP_{i,t}=\beta_{1}+\beta_{2}DF_{i,t}+\gamma Control_{i,t}+\lambda_{t}+\upsilon_{i}+\varepsilon_{i,t}$$

$CCEP_{i,t}$ denotes the urban carbon emission performance and $DF_{i,t}$ denotes the digital finance. $Control_{i,t}$ denotes the number of control variables including population density (Popden), infrastructure (Infra), GDP per capita (Pgdp), government expenditure (Ge), urbanization (Urban), afforestation coverage (Green), economic growth (G*r*), industry structure (Sec), and environment regulation (Envir). $\upsilon_{i}$ and $\lambda_{t}$ are used for controlling the fixed effect of city and year, respectively. $\varepsilon_{i,t}$ is the random error term.

### 3.3. Data Description

Our samples include 281 prefecture-level cities from China’s mainland (all the prefecture-level cities in Tibet are excluded because of data availability). Our data began in 2011 as the data about the digital finance index are available only after that, and our data ended in 2019 as more recent data are incomplete.

Because this paper focuses on the analysis of urban CCEP, following Yang, et al. [55] and Wang and Feng [56], we chose labor, capital stock, and energy consumption as input variables, and chose economic outputs and CO_2_ emissions as desirable outputs and undesirable outputs, respectively. Labor is the number of employees in each city. Gross domestic product is chosen as a desirable output variable and converted into 2003 constant prices using the consumer price index. Based on the yearly fixed-asset investment data, we estimated the capital stock with the perpetual inventory method by taking 2003 as the base year [57]. The energy input was measured by the consumption of electricity. Based on the methods of Andres, et al. [58] and Oda, et al. [59], we aggregated and corrected the raster data of CO_2_ emission, which are from the Open-Data Inventory for Anthropogenic Carbon dioxide, to obtain the carbon emission data of China’s cities during 2011–2019. The above variables are all from the *China City Statistical Yearbook.* The statistics of DEA variables are listed in Table 1.

In the regression analysis, due to the use of the global production technology set, we used the urban carbon emission performance as the explained variable. To ensure the robustness of the regression results, referring to Yan, et al. [60], we first used the accumulative change of carbon emission performance as the explained variable (set 2011 as the base year and its accumulative change is 1, then the accumulative change value in year t is $Cep_{i,t}/Cep_{i,2011}$). Secondly, the CCEP calculated based on the standard SBM model and urban CO_2_ emission intensity is further used as explained variables for robustness tests, respectively. The digital finance index (DF) is from the IDF (Institute of Digital Finance) of Peking University. According to the forms and characteristics of digital financial services, digital finance includes the following three subdivision indicators: namely, coverage breadth (DF-CB), usage depth (DF-UD), and degree of digital support services (DF-DSS) [61]. The coverage breadth was calculated by the number of electronic accounts, which represents the population covered by digital finance. The usage depth was measured based on the actual use frequency of internet financial services. For DF-DSS, the index focuses on reflecting the convenience and efficiency of digital finance. Meantime, the DF index and its three sub-indicators all showed significant growth from 2011 to 2019, indicating that Chinese digital finance has developed rapidly, and the coverage breadth, depth of use, and service efficiency of digital finance have been significantly improved.

To reduce the estimation bias due to omitted variables, we added the control variables as described in Section 3.2 to the regression model. We first controlled for population density (Popden), urbanization rate (Urban), economic growth (Gr), and economic development level (Pgdp) as they could influence environmental efficiency through scale effects [62,63]. Then, we controlled for the proportion of secondary industry (Sec) and afforested rate (Green) as the secondary-industry share is more carbon-intensive [44], while afforested areas could absorb carbon emissions. We finally controlled for infrastructure (Infra), government expenditure (Ge), and environmental regulations (Envir) as they are influencing factors of the energy intensity [5,64]. The definition of the control variables is listed in Table A1. The data of control variables are all obtained from the *China City Statistics Yearbook* and *China’s National Bureau of Statistics*.

In addition, to further alleviate the potential endogeneity in the regression model, this paper uses the urban central geographic distance between the sample city and Hangzhou as the instrumental variable. Firstly, Hangzhou is the pioneer and benchmark city of China’s digital development and digital finance development, with a large number of talents and the perfect infrastructure required. Secondly, the headquarters of China’s influential Internet companies are mainly set up in Hangzhou (such as Alibaba), whose digital finance development index ranked first in China in 2019, at 321.64. Therefore, the development of digital finance in Hangzhou has an obvious demonstration and leading role for other cities in China. Considering the significant spillover and diffusion effects of urban financial development [65,66], the development of digital finance in cities close to Hangzhou will be significantly improved, hence instrumental variables and explanatory variables have obvious correlation characteristics. Moreover, geographical distance as an objective index will not significantly affect the comprehensive carbon emission performance level of cities. Therefore, the index meets the relevant assumptions of instrumental variables in theory.

Furthermore, as discussed in the theoretical analysis, digital finance may further improve CCEP by promoting urban green innovation (Green-Inno) and urban tertiary industry development (DTI). Therefore, we chose the green patent applications per capita and the proportion of output value of the tertiary industry to further explore the impact path of digital finance. Finally, we used financial development (FD), measured by the ratio of loan balances to GDP [50], to conduct the cross-tests.

The statistics of regression variables are listed in Table 2.

## 4. Results and Discussion

Firstly, in Section 4.1 we analyze China’s urban CCEP and its change characteristics during 2011–2019. Then, we empirically analyze the impact of digital finance on CCEP in Section 4.2. We make further efforts to discuss the robustness and endogeneity of the regression results in Section 4.3. In Section 4.4 we conduct the channel tests, and further conduct the cross tests according to heterogeneity of urban development characteristics.

### 4.1. The Dynamic Change Characteristics of Urban CCEP

**Figure 1:**
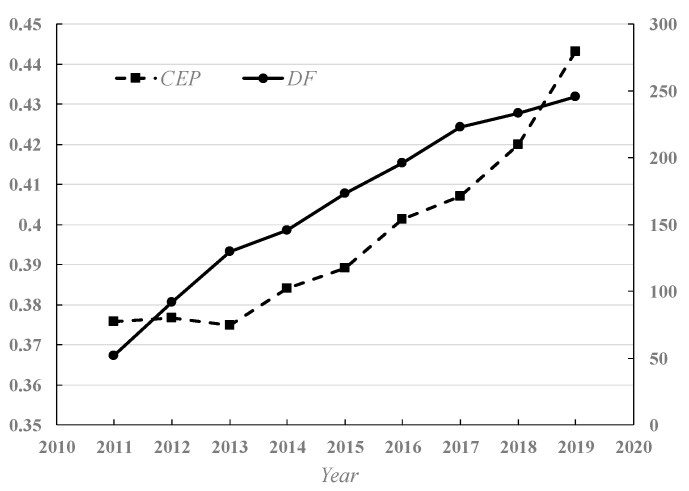
Average carbon emission performance and digital finance index of China’s cities from 2011 to 2019.

**Figure 2:**
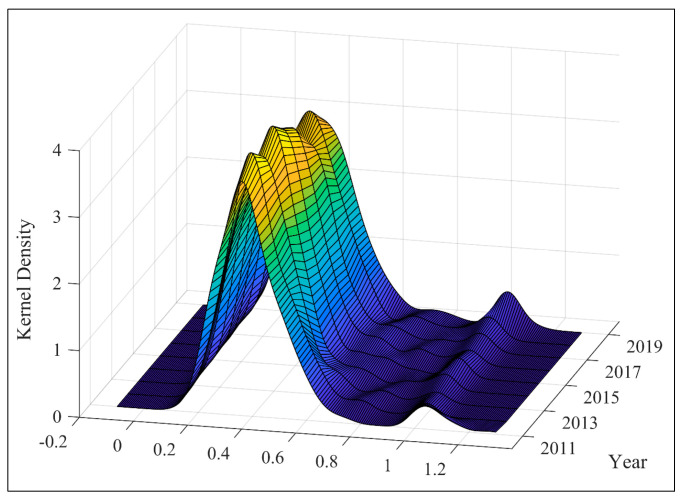
The kernel density distribution of Urban CCEP from 2011 to 2019.

Figure 1 illustrates the average performance and average financial development index of Chinese cities from 2011 to 2019. Figure 2 represents the Kernel density distribution of China’s urban carbon performance from 2011 to 2019. The average carbon emission performance of the sample cities is calculated by the weighted average method [67]. As shown in Figure 1, the average CCEP of Chinese cities shows an upward trend from 0.375 in 2011 to 0.445 in 2019. Simultaneously, the average digital finance development of the sample cities also shows an upward trend, which is consistent with the average CCEP. In addition, as shown in Figure 2, the distribution peak value of urban CCEP gradually moves from low-value areas to high-value areas from 2011 to 2019. Specifically, in 2019, the distribution frequency of China’s urban CCEP in the high-value areas increases largely and shows a trend of intensifying convergence. The above results also reflect that the urban CCEP of most Chinese cities has been significantly increased in 2019 compared with 2011.

### 4.2. The Impact of Digital Finance on CCEP

To explore the impact of digital finance on China’s urban carbon emission performance, we firstly adopt different panel regression specifications to estimate the basic model shown in formula (3). The panel regression results using the fixed effect (FE), the Tobit, and the random effect (RE) models, respectively, are presented in Table 3. As shown in Table 3, when the FE model is used for estimation (shown in columns (1) and (2)), regression coefficients of DF are significantly positive at the 1% statistical level. The results indicate that digital finance can significantly improve China’s urban CCEP. Specifically, the regression coefficient of DF in column (2) is 0.00196, indicating that when the index of digital finance increases by 100, the urban comprehensive carbon performance increases by 0.196. In addition, when the Tobit and RE are adopted (shown in columns (3) and (4)), the regression coefficients of DF are still positive at the 1% statistical level and there is a small variation of regression coefficients between different regression specifications.

Besides, to alleviate the potential contemporaneous reverse causality and fully consider the lag impact of the DF index on China’s CCEP, the one phase lag of DF (L.DF) is chosen as the core explanatory variable. The coefficient of L.DF is significantly positive with the specifications of the FE, the Tobit, and the RE in columns (1) to (3) of Table 4, which also indicates that, after considering the contemporaneous reverse causality and lag effect, digital finance still significantly promotes the CCEP. Therefore, under the goal of low-carbon development, Chinese governments should proceed to promote the development of the digital financial system, which could provide more financial support for economic entities to carry out green and low-carbon economic activities.

To explore the impact of different dimensions of digital finance on urban CCEP, we further incorporate each sub-indicator (namely, DF-CB, DF-UD, and DF-DSS) into the regression model. Specifically, for coverage breadth, considering the scale effect, we add the quadratic term of DF-CB into the regression model. It can be found, in the second column of Table 5, that the coefficient of DF-CB is significantly negative, while the coefficient of DF-CB^2^ is significantly positive, indicating that the impact of coverage breadth on urban CCEP shows a U-shaped trend. The result also indicates that only when the number of people and enterprises involved in digital financial services reaches a certain scale, the coverage breadth could effectively improve the carbon emission performance. Otherwise, some financial capital is separated from the traditional financial platform, resulting in the insufficient use of funds, which causes a certain inhibitory effect. For the use depth, as shown in Table 5, the regression coefficients of DF-UD and L.DF-UD are both significantly positive, indicating that deepening the usage of digital finance could improve the urban comprehensive carbon emission performance. The use depth reflects the diversification and multi-level of the digital finance development. With the deepening use of digital finance, various digital financial services (such as credit and insurance services) are continuously improved, which can provide more funds for entrepreneurs and enterprises, and reduce the risk of green and low-carbon innovation. For the degree of digital support services, the regression coefficients of DF-DSS are significantly positive in Table 5. Occurring to the improvement of DF-DSS making financial services more efficient and transactions more conveniently, the transaction costs of economic entities have been reduced and financial resources have been more fully allocated, which further improves the performance of urban carbon emission.

### 4.3. Robustness and Endogeneity Tests

In the above discussion, we have ensured the robustness of the results by using different regression specifications and adopting the lag term of core explanatory variables. In this section, we further explore the robustness of regression results with the method of replacing core explained variables. Firstly, we confer to the practice of Lee and Lee [50], using the accumulative change of comprehensive carbon emission performance as the explained variable (the ratio of carbon emission performance in year *t* to that in 2011). Secondly, based on the SBM model, we evaluate the value of carbon emission performance and take it as the explained variable. In addition, considering that the Chinese government has always been chasing the goal of reducing regional carbon emission intensity to achieve low-carbon development, hence we integrate urban CO_2_ emission intensity into the regression model. Table 6 shows the relevant results.

As shown in columns (1) and (3) of Table 6, whether it is the accumulative change value or SBM performance, the coefficients of DF are both significantly positive. In the meantime, considering the lag effect, in columns (2) of Table 6, the coefficient of L.DF is also significantly positive at the 5% statistical level. For the CO_2_ emission intensity, as shown in columns (5) and (6) of Table 6, the regression coefficients of DF and L.DF are both significantly negative, indicating that the digital finance has effectively reduced the regional CO_2_ emission intensity. Therefore, the above results comprehensively reflect that digital finance plays a positive role in realizing the urban low-carbon development, and the empirical results have a strong robustness.

In order to effectively alleviate the potential endogenous issues and identify the causal effect of China’s digital finance development on CCEP, this paper introduces geographical distance from each sample city to Hangzhou as an instrumental variable for regression test. It should be emphasized here that the distance variable does not change with time; therefore, we use this variable to interact with the time trend to include the time factor. As mentioned above, the digital financial development in Hangzhou has an obvious demonstration and leading role for other cities. Therefore, the variable of geographical distance from Hangzhou can meet the relevant assumptions of instrumental variables. The regression results are shown in Table 7. In the first stage, the value of the Kleibergen–Paap rk F statistic is 117, which is far greater than the critical value of the Stock–Yogo test. In addition, the null hypothesis that instrumental variables are weakly correlated is rejected. In the meantime, the regression coefficient of IV in the first stage is significantly negative, indicating that the development of digital finance in the sample cities drops with the increase in distance. As shown in the second stage, the regression coefficient of DF is significantly positive. The above results also indicate that after eliminating the potential endogeneity, digital finance still has a positive impact on urban CCEP, and the regression result is robust as before.

### 4.4. Channel Tests and Heterogeneity Analysis

In order to explore that digital finance can improve urban CCEP by promoting urban green technology innovation and developing the tertiary industry, we further introduce Green-Inno and DTI variables into the regression model. The results of the channel tests are shown in Table 8. As shown in columns (1) to (3), the regression coefficients of DF are significantly positive under the pooled ordinary least squares (Pols), the RE, and the FE model, which are consistent with the previous research results [24,26]. The results show that digital finance could promote regional green innovation, and hypothesis I is verified. Secondly, we explore the impact of digital finance on the urban tertiary industry. As shown in columns (4) to (6) of Table 8, for DTI, the regression coefficient of DF is significantly positive when using the Pols, the RE, and the FE models, respectively, indicating that digital finance significantly promotes the development of the urban tertiary industry, hence hypothesis II is verified. Therefore, according to the above empirical results of channel tests, we can get that urban digital finance mainly improves CCEP by promoting green innovation and the development of the tertiary industry.

As the impact of digital finance on urban CCEP may vary in different regions, we group the sample cities based on the degree of traditional financial development, differences in environmental regulation policies and economic development characteristics. For traditional finance, sample cities are divided into two types according to the median urban financial development level, namely the high financial development group (High-FD) and the low financial development group (Low-FD). In addition, according to whether it belongs to the two-control zone (controlling acid rain and SO2 emissions), the sample cities are further divided into a two-control zone (TCZ) and a non-two-control zone (Non-TCZ). Besides, considering the characteristics of urban economic development, we divide the sample cities into two groups (old industrial base cities (OIB) and non-old industrial base cities (Non-OIB)) according to whether the cities belong to old industrial bases. The relevant heterogeneity results are shown in Table 9.

In columns (1) and (2), no matter in High-FD cities or in Low-FD cities, the regression coefficients of DF are both positive with values of 0.00239 and 0.00094, respectively. The results show that no matter in a High-FD city or a Low-FD city, digital finance can boost their carbon emission performance, and its positive effect is stronger in cities with better traditional financial development. This is mainly because areas with more developed traditional finance provide a better foundation for the growth of digital finance. For example, financial institutions can provide diversified financial services, and enterprises have strong financial thoughts. Therefore, in such areas, digital finance gets stronger development support, and innovative enterprises will also get more financial services, so the positive promotion effect is more obvious.

Secondly, as shown in columns (3) and (4) of Table 9, in non old industrial areas, digital finance significantly promotes the regional CCEP, while in old industrial cities, the positive effect is not significant. The possible explanation is that due to the high proportion of heavy industry output in China’s old industrial cities, the green transformation of industries is relatively difficult, and the urban innovation capabilities and service industry development are poor, so digital finance cannot play an effective role in promoting.

Finally, considering the differences in environmental policies, we further explore the synergy between digital finance and environmental regulation policy. As shown in columns (5) and (6) of Table 9, the DF regression coefficient of cities in the two-control zone is positive with the value of 0.00233, reflecting that the index of digital finance in the two-control zone increases by 100, the comprehensive carbon emission performance increase by 0.233. However, in the Non-TCZ, the regression coefficient of DF is not significant. The above results reflect that there is a positive synergy relationship between digital finance and the government’s environmental control policies. Due to that there are stronger environmental control requirements in the two-control zone, enterprises must carry out green innovation to reduce the emission of pollutants such as sulfur dioxide, and the development of digital finance could provide diversified financial services to meet the capital needs of economic entities.

## 5. Conclusions and Policy Implications

Controlling CO_2_ emission has been an urgent job for the whole world facing climate change. As a major carbon emitter, China has proposed the goals of achieving a carbon peak and carbon neutrality. Considering that there are strong relationships among economic development, carbon emissions, and energy consumption, promoting urban comprehensive carbon emission performance could contribute to realizing the CO_2_ emissions reduction, and it is also the inevitable choice for China’s low-carbon development. Meanwhile, in recent years, relying on new technologies, digital finance improves people’s well-being by providing more inclusive financial support. However, few pieces of literature are contributing to exploring the relationship between digital finance and urban low-carbon development. Therefore, to identify the economic consequences of digital financial development, and to systematically explore the impact of digital financial development on urban CCEP, based on data of 281 cities from 2011 to 2019 in China, this paper empirically identifies the casual relationship between digital finance and urban low-carbon development after accurately assessing Chinese urban comprehensive carbon emission performance. In addition, we further conduct a set of robustness tests and address the endogeneity issue. Finally, we explore the channels of digital finance affecting urban carbon emission performance and conduct heterogeneity tests based on urban development characteristics.

The results show that, from 2011 to 2019, the average CCEP of China’s cities shows an upward trend, and the increase in the eastern area is more obvious, suggesting that urban economic activities tend to be more efficient, highly productive, and produce less CO_2_ emissions. Secondly, the regression results suggest that digital finance significantly improves urban carbon emission performance. Meantime, for the different sub-indicators, there is a U-shaped relationship between the coverage breadth and carbon emission performance, reflecting that when the coverage breadth reaches a certain scale, the urban carbon emissions performance can be effectively improved, and the deepening usage of digital finance and the improvement of digital support services could significantly improve CCEP. In addition, after conducting a set of robustness tests and addressing the endogeneity issue, the above conclusion is robust. Thirdly, through channel tests, we find that digital finance mainly improves urban CCEP by promoting green innovation and the development of the urban tertiary industry. Finally, cross-tests show that digital finance has a stronger impact on improving carbon emission performance in High-FD cities, and the positive effect is significant in non-old industrial base cities and the two-control zone.

Our research provides some enlightenment for China’s low-carbon sustainable development planning and environmental policy. From the perspective of macro mechanism, the government should continuously promote the healthy growth of digital finance and accelerate the perfection of the digital financial system. Specifically, the government should firstly focus on accelerating the construction of digital infrastructure, improving the coverage breadth, and increasing the development scale of digital finance. Secondly, the government needs to improve urban digital financial functions and services, and realize the potential of digital finance in payment, credit, insurance, and other aspects. Thirdly, the government should vigorously support the integration of technology and finance and improve the service efficiency. For microeconomic entities, the government should pay attention to guiding the flow of financial funds. On the one hand, digital financial funds should be invested in innovative enterprises to promote green technology innovation. On the other hand, it is worthy to attach importance to allocating digital financial resources into the service industry to promote the development of the service industry. Considering the heterogeneity of urban development characteristics, the central government should draft differentiated digital finance development strategies. For cities with more developed traditional finance, we should fully recognize the positive emission reduction effect of digital finance. As most eastern coastal cities and developed cities have a high level of financial development and relatively large CO_2_ emissions, hence promoting the development of digital finance in the above regions could further achieve more positive effects in terms of promoting the low-carbon development. Finally, the government should grasp the development characteristics of digital finance and recognize the synergy between digital finance and the government’s environmental control policies, to help the relevant environmental policies achieve good environmental governance effects.

Although this paper fills the gap in the extant research on how digital finance affects the urban carbon emission performance, there are still some limitations. First, whether it is the urban digital finance development or urban carbon emission performance, there is a possibility of spatial correlation, that is, the local development of digital finance (or local carbon emission performance) can significantly influence the development of digital finance (or carbon emission performance) in surrounding cities. Therefore, in future research, considering the characteristics of spatial correlation can provide a more realistic and meaningful reference for urban low-carbon development. Secondly, although the city-level data are used, the data in this paper are still based on the macro level. Considering that the digital finance can significantly boost green innovation, therefore, exploring the impact of digital finance on enterprise green innovation and enterprise pollutant emission based on microenterprise data can further analyze the economic consequences of the digital finance development and offer a valuable reference for promoting the development of digital finance.

## Figures and Tables

**Table 1:** Summary statistics of DEA variables.

Variable	Unit	Obs	Min	Max	Mean	Std. Dev
Energy consumption	10^8^ KWh	2529	6.61	1568.58	179.53	203.17
Capital stock	10^8^ RMB	2529	375.59	77,049.69	6607.05	7412.50
Labor	10^4^ persons	2529	11.31	1729.08	123.04	165.25
Gross domestic production	10^8^ RMB	2529	53.92	24,833.72	1798.73	2334.06
CO_2_	10^5^ tons	2529	12.21	2801.32	324.30	338.01

**Table 2:** Summary statistics of regression variables.

Variable	Obs	Min	Max	Mean	Std. Dev
Digital finance	2529	17.02	321.64	165.58	65.38
DF-CB	2529	1.88	310.91	155.91	63.34
DF-UD	2529	4.29	331.96	163.35	67.97
DF-DSS	2529	2.70	581.23	201.58	81.93
CCEP	2529	0.0935	1.2781	0.3983	0.1695
Popden *	2529	2.2771	7.9226	5.7632	0.8882
Infra *	2529	2.8969	8.8962	5.8305	0.8839
Pgdp *	2529	3.8291	8.2119	5.7375	0.6880
Ge	2529	0.0438	0.9154	0.1992	0.1013
Urban	2529	0.0035	0.9997	0.5449	0.1507
Green	2529	0.0059	0.9525	0.3972	0.0691
Gr	2529	−0.1938	0.2396	0.0854	0.0380
Sec	2529	0.1171	10.5763	0.4722	0.3358
Envir	2525	0.0002	0.0181	0.0058	0..0023
Green-Inno	2520	0.0000	26.8175	0.7244	1.7625
DTI	2520	0.1808	0.7213	0.4171	0.1001
Accumulated change	2529	1.6679	2.7086	0.9688	0.2268
SBM performance	2529	0.0935	1.0000	0.3974	0.1663
Distance	2520	0.0000	3445.13	1029.40	551.07
FD	2528	0.1179	9.6228	0.9866	0.6176

Note: Symbol of * in this table denotes that the relevant variables have been logarithmically processed.

**Table 3:** Estimate results of the benchmark model.

Variables	(1)	(2)	(3)	(4)
	FE	FE	Tobit	RE
DF	0.00147 ***	0.00196 ***	0.00217 ***	0.00216 ***
	(2.62)	(2.83)	(7.47)	(3.17)
Popden		0.0463	0.0084	0.0079
		(0.61)	(0.76)	(0.53)
Infra		−0.0126	−0.0034	−0.0028
		(−1.36)	(−0.55)	(−0.32)
Pgdp		−0.1338 ***	−0.0750 ***	−0.0703 **
		(−3.25)	(−5.40)	(−2.40)
Ge		−0.2466 **	−0.1237 **	−0.1121
		(−2.25)	(−2.22)	(−1.18)
Urban		−0.1252	−0.0340	−0.0306
		(−1.41)	(−0.73)	(−0.46)
Green		−0.1300 ***	−0.1409 ***	−0.1420 ***
		(−3.13)	(−4.48)	(−3.35)
Gr		0.2604 ***	0.1821 ***	0.1760 ***
		(3.57)	(3.00)	(2.81)
Sec		−0.0085 **	−0.0100 *	−0.0102 ***
		(−2.17)	(−1.90)	(−3.29)
Envir		−2.0676 *	−2.5472 ***	−2.6010 **
		(−1.74)	(−2.85)	(−2.19)
Constant	0.1537	0.8110 *	0.7840 ***	0.7559 ***
	(1.65)	(1.95)	(8.06)	(4.37)
City effect	Yes	Yes	Yes	Yes
Year effect	Yes	Yes	Yes	Yes
Observations	2529	2525	2525	2525
R-squared	0.812	0.824	—	0.091
Number of cities	281	281	281	281

Note: T statistics are in parentheses. Symbols of ***, **, and * denote 1%, 5%, and 10% significance levels, respectively. The robust standard errors are used in the regression. Unless otherwise stated, the following tables are consistent with this table.

**Table 4:** The lag effect of DF index.

Variables	(1)	(2)	(3)
	FE	Tobit	RE
L.DF	0.00084 *	0.00113 ***	0.00115 **
	(1.79)	(4.32)	(2.38)
Constant	0.4081	0.5338 ***	0.5118 ***
	(1.07)	(5.73)	(3.36)
Control variables	Yes	Yes	Yes
City effects	Yes	Yes	Yes
Year effect	Yes	Yes	Yes
Observations	2244	2244	2244
R-squared	0.879	—	0.067
Number of cities	281	281	281

Note: Control variables indicates that the control variables have been added to the regression model, and the lag terms of some control variables are used in this regression. Symbols of ***, **, and * denote 1%, 5%, and 10% significance levels, respectively.

**Table 5:** Coverage breadth, digital support services, and usage depth.

Variables	(1)	(2)	(3)	(4)	(5)	(6)
	FE	FE	FE	FE	FE	FE
DF-CB	−0.00006	−0.00408 ***				
	(−0.06)	(−3.57)				
DF-CB^2^		9.29 × 10^−6^ ***				
		(7.69)				
DF-UD			0.00098 ***			
			(2.69)			
L. DF-UD				0.00038 *		
				(1.73)		
DF-DSS					0.00043 ***	
					(3.97)	
L. DF-DSS						0.00019 **
						(2.38)
Constant	0.7138	1.3774 ***	0.6816	0.3749	0.7728	0.3065
	(1.38)	(3.08)	(1.35)	(0.97)	(1.45)	(0.81)
Control variables	Yes	Yes	Yes	Yes	Yes	Yes
City effect	Yes	Yes	Yes	Yes	Yes	Yes
Year effect	Yes	Yes	Yes	Yes	Yes	Yes
Observations	2525	2525	2525	2244	2525	2244
R-squared	0.820	0.844	0.823	0.879	0.823	0.879
Number of cities	281	281	281	281	281	281

Symbols of ***, **, and * denote 1%, 5%, and 10% significance levels, respectively.

**Table 6:** Robustness: alternative indicator of carbon emission performance.

Variables	(1)	(2)	(3)	(4)	(5)	(6)
	Accumulated Change		SBM Performance		CO_2_ Emission Intensity	
	FE	FE	FE	FE	FE	FE
DF	0.00358 ***		0.00191 ***		−0.00034 **	
	(3.36)		(2.96)		(−2.04)	
L.DF		0.00195 **		0.00078		−0.00031 **
		(2.26)		(1.61)		(−2.02)
Control variables	Yes	Yes	Yes	Yes	Yes	Yes
	(−2.50)	(−2.69)	(−1.89)	(−2.31)	(−1.96)	(−1.41)
Constant	1.8524 **	1.0912	0.7740 *	0.3393	0.1984	0.2312
	(2.05)	(1.36)	(1.66)	(0.93)	(0.57)	(0.64)
City effect	Yes	Yes	Yes	Yes	Yes	Yes
Year effect	Yes	Yes	Yes	Yes	Yes	Yes
Observations	2525	2244	2525	2244	2525	2244
R-squared	0.665	0.743	0.831	0.881	0.983	0.981
Number of cities	281	281	281	281	281	281

Symbols of ***, **, and * denote 1%, 5%, and 10% significance levels, respectively.

**Table 7:** IV-2SLS regression.

Variables	(1)	(2)
	Fixed Effect	
	First-stage	Second-stage
DF		0.00829 ***
		(4.47)
IV: Distance	−0.00110 ***	
	(−10.82)	
Popden	21.7361 *** (5.42)	−0.1355 * (−1.75)
Infra	0.4678 (1.27)	−01378 (−1.60)
Pgdp	6.6810 *** (5.15)	−0.1919 *** (−6.89)
Ge	−11.3879 ** (−2.31)	−0.1175 (−1.27)
Urban	−3.1302 (−0.55)	−0.1056 ** (−1.98)
Green	3.8904 * (1.74)	−0.1440 *** (−3.68)
Gr	−18.0013 *** (−2.75)	0.3492 *** (4.77)
Sec	−0.3329 (−0.98)	−0.0034 (−0.57)
Envir	−66.3621 (−1.16)	−1.3849 (−1.44)
City effect	Yes	Yes
Year effect	Yes	Yes
Cluster	Yes	Yes
Observations	2516	2516
Kleibergen–Paap rk LM statistic	120.55 [0.0000]	—
Kleibergen–Paap Wald rk F statistic	117.00 <16.38>	—
Number of cities	281	281

Note: The numbers within < > are the critical values of the Stock–Yogo test at the 10% significant level. The numbers in [] are the *p* value of the corresponding test statistic. Symbols of ***, **, and * denote 1%, 5%, and 10% significance levels, respectively.

**Table 8:** Channel tests.

Variables	(1)	(2)	(3)	(4)	(5)	(6)
	Green-Inno			DTI		
	Pols	RE	FE	Pols	RE	FE
DF	0.0286 ***	0.0433 ***	0.0315 ***	0.00238 ***	0.00074 ***	0.00029 *
	(10.94)	(14.36)	(9.82)	(15.07)	(4.90)	(1.87)
Popden	0.5457 ***	0.4565 ***	7.0020 ***	0.0130 ***	0.0160 ***	−0.1061 ***
	(15.50)	(6.13)	(15.83)	(6.11)	(3.40)	(−4.84)
Infra	−0.1769 ***	−0.1704 ***	−0.2384 ***	0.0096 ***	0.0076 **	−0.0023
	(−3.62)	(−2.72)	(−3.49)	(3.27)	(2.38)	(−0.68)
Pgdp	1.6251 ***	0.9631 ***	0.1990	−0.0010	−0.0308 ***	−0.0779 ***
	(18.15)	(7.51)	(1.26)	(−0.18)	(−4.58)	(−9.98)
Ge	7.6482 ***	2.7098 ***	−0.2061	0.3868 ***	0.2000 ***	0.0727 **
	(21.00)	(5.00)	(−0.33)	(17.52)	(7.08)	(2.37)
Urban	−0.3220	−2.0774 ***	−4.6118 ***	0.0421 **	0.1212 ***	0.0358
	(−1.07)	(−4.64)	(−8.32)	(2.30)	(5.13)	(1.31)
Envir	−37.6384 ***	−44.6059 ***	−37.0258 ***	−0.0334	1.5061 ***	1.4775 ***
	(−3.26)	(−4.57)	(−3.95)	(−0.05)	(3.18)	(3.20)
Constant	−13.3010 ***	−8.1781 ***	−38.9106 ***	0.0248	0.2538 ***	1.3599
	(−27.36)	(−10.44)	(−14.30)	(0.84)	(5.71)	(10.09)
City effect	No	Yes	Yes	No	Yes	Yes
Year effect	Yes	Yes	Yes	Yes	Yes	Yes
Observations	2516	2516	2516	2516	2516	2516
R-squared	0.518	0.255	0.343	0.447	0.643	0.663
Number of cities	280	280	280	281	281	281

Symbols of ***, **, and * denote 1%, 5%, and 10% significance levels, respectively.

**Table 9:** Cross-sectional test: environmental regulation, old industry bases, and two-control zone.

Variables	(1)	(2)	(3)	(4)	(5)	(6)
	High-FD	Low-FD	OIB	Non-OIB	TCZ	Non-TCZ
	FE	FE	FE	FE	FE	FE
DF	0.00239 **	0.00094 *	−0.00037	0.00302 ***	0.00233 ***	0.00119
	(2.05)	(1.90)	(−0.37)	(4.20)	(4.08)	(1.16)
Constant	0.5152	2.2255 **	1.3814	1.0426	−0.0405	2.6739 ***
	(0.88)	(2.54)	(1.29)	(1.50)	(−0.07)	(3.45)
Control Variables	Yes	Yes	Yes	Yes	Yes	Yes
City effect	Yes	Yes	Yes	Yes	Yes	Yes
Year effect	Yes	Yes	Yes	Yes	Yes	Yes
Observations	1245	1253	851	1674	1422	1103
R-squared	0.783	0.886	0.818	0.830	0.8514	0.821
Number of cities	170	175	95	186	158	123

Symbols of ***, **, and * denote 1%, 5%, and 10% significance levels, respectively.

## Data Availability

The data presented in this study are available on request from the corresponding author.

## Appendix A

**Table A1:** The definition of control variables.

Variable	Definition
Popden	Total population/total area (taking log)
Infra	Road area/total population (taking log)
Pgdp	GDP/total population (taking log)
Ge	Fiscal expenditure/GDP
Urban	Urban population/total population
Green	Afforested area/total area
Gr	The growth rate of GDP
Sec	The added value of second industry/GDP
Envir	The proportion of text that is related to environmental production and emissions in cities’ government work reports
